# Artificial intelligence, robotics and eye surgery: are we overfitted?

**DOI:** 10.1186/s40942-019-0202-y

**Published:** 2019-12-16

**Authors:** Müller G. Urias, Niravkumar Patel, Changyan He, Ali Ebrahimi, Ji Woong Kim, Iulian Iordachita, Peter L. Gehlbach

**Affiliations:** 1 Wilmer Eye Institute, Johns Hopkins Hospital, Baltimore, MD 21287 USA; 2 Federal University of Sao Paulo, São Paulo, 04023-062 Brazil; 3 Laboratory for Computational Sensing and Robotics, Johns Hopkins University, Baltimore, MD 21218 USA; 4 School of Mechanical Engineering and Automation at, Beihang University, Beijing, 100191 China; 5 Whiting School of Engineering, Johns Hopkins University, Baltimore, MD 21218 USA

**Keywords:** Robotic surgical procedures, Robotics, Artificial intelligence, Retina, Ophthalmology

## Abstract

Eye surgery, specifically retinal micro-surgery involves sensory and motor skill that approaches human boundaries and physiological limits for steadiness, accuracy, and the ability to detect the small forces involved. Despite assumptions as to the benefit of robots in surgery and also despite great development effort, numerous challenges to the full development and adoption of robotic assistance in surgical ophthalmology, remain. Historically, the first in-human–robot-assisted retinal surgery occurred nearly 30 years after the first experimental papers on the subject. Similarly, artificial intelligence emerged decades ago and it is only now being more fully realized in ophthalmology. The delay between conception and application has in part been due to the necessary technological advances required to implement new processing strategies. Chief among these has been the better matched processing power of specialty graphics processing units for machine learning. Transcending the classic concept of robots performing repetitive tasks, artificial intelligence and machine learning are related concepts that has proven their abilities to design concepts and solve problems. The implication of such abilities being that future machines may further intrude on the domain of heretofore “human-reserved” tasks. Although the potential of artificial intelligence/machine learning is profound, present marketing promises and hype exceeds its stage of development, analogous to the seventieth century mathematical “boom” with algebra. Nevertheless robotic systems augmented by machine learning may eventually improve robot-assisted retinal surgery and could potentially transform the discipline. This commentary analyzes advances in retinal robotic surgery, its current drawbacks and limitations, and the potential role of artificial intelligence in robotic retinal surgery.

## Article

Artificial intelligence was a term coined in 1955 by McCarthy et al. in a workshop proposal for the following year, to be attended at Dartmouth College [1]. The meeting was to be a collaborative effort aimed at developing machines that could not only use a language, but design concepts and solve problems. In 1988, the first robot-assisted procedure was published by Kwoh [2], a robot-guided brain biopsy. In some ways the concept of machine assistance and autonomous task performance had occurred well prior to McCarthy’s proposal. The birth of mathematical logic, highlighted by such works as e.g., *Principia Mathematica* in 1912 [3], bolstered thoughts that had occurred throughout the 17th century that applied systematic algebra could ultimately reproduce human thinking, and that this substitution could lead to automated thought. This scientific “boom” inspired latter period science fiction movies, but most importantly many researchers of the time were excited to dedicate their resources and research efforts to these ideas.

By way of example, in 1950 Alan Turing published a then distinguished article [4], some years before the term ‘artificial intelligence’ was first used, and many years before the first robot-assisted clinical procedure was published. In Turing’s paper, he suggested that machines could reproduce human thoughts, and this assertion was based on the principle that human decisions were based on available information. He concluded that this hypothesis could be tested and verified if the machine decision was nearly indistinguishable from the human decision. This concept and its further development, in conjunction with advances in technology, certainly raised concerns for machine–human substitution and stimulated discussion around the question of what would be the eventual role of machinery in decision-making tasks [5–7]. Despite whatever concerns existed, early technology development released humans from simple and repetitive tasks. Now decades later, humanity is faced with integrating such advanced technology as self-driving cars [8], FDA-approved surgical robots [9] and AI systems that appear human, in a scaring and “Turing-provocative” way [10]. As many of the technological concepts being developed were designed around human thinking architecture [11], advances in artificial intelligence were also based on the human cortical systems and with relation to the visual pathways [12].

All inspiration and optimism aside, the role of robotic assistance in modifying outcomes in ophthalmology is evolving. However, there remain significant obstacles to consolidate robotics in medicine. These include, but are not limited to, implementation costs, safety concerns, questionable efficiency and unproven efficacy to name a few [13–15]. With regards to retinal robotic surgery, obstacles affect to an even greater extent due to the delicate, fragile, transparent, unforgiving, nonregenerative and micron-scale of the target tissue, not to mention the early stage of development of such robots at this time [16–18]. With due consideration for the uniquely fragile retinal tissue, most procedures in the constrained intraocular environment demand exceedingly high dexterity, concentration and tremor control leading to questions as how recent advances in robotics and artificial intelligence might prove beneficial while at the same time identifying in which opportunities this technology could be applied.

Human accuracy in retinal microsurgery is reported to be at best between 20 and 40 µm in its lower bound [19], as average human tremor is noted to be approximately 100 µm in its peak-to-peak excursion [20]. Likewise the average human threshold for a tactile perception is reported to be approximately 7.5 mN [21] which coincidently is the force reported in prior work to be sufficient to cause a tear in the retina of a rabbit. [22]. In such a scenario, the robot’s stability, sturdiness and precision might be exceedingly useful and delicate intraocular procedures, e.g. membrane peeling, subretinal treatments and vein cannulation, could benefit from such systems. In 1989 the first robotic assistance in ophthalmology was published by Guerrouad and Vidal [23]. This in turn encouraged further studies in areas from force sensitive instruments [24, 25] to robotic platform development [26–28], and some examples of research centers are at Johns Hopkins University, Katholieke Universiteit Leuven, Oxford University and University of California.

Despite these advances, many challenges remain including but not limited to reducing instrument size and cost, implementing fast data processing and adapting systems to smaller work environments. It was not until 2018 (18 years after da Vinci robot approval for laparoscopic use), that the first human study with an eye robot was published [29]. Although these provided promising results, other questions on safety and efficiency, not to mention cost-remain. Although they are able to be used during the entire vitrectomy procedure, the loss of instrument awareness and limitations on robot’s end-effector velocity and tilting angle lean their usage to be tasks-specific and the macula to be a preferable target site. Emerging directions in instrument awareness and research development include force sensing instruments [24, 30]—with present generation tools utilizing optical sensors. Robots equipped with force sensing capabilities are intriguing in part due to their potential to enhance safety, efficacy and functionality, as well as to provide information feedback from the robotic tool in use, in order to improve function and utility [31]. At present, the robotic platform alone does not replicate the surgical free-hand experience, but further development of the robotic tool, including enhanced sensor input and incorporation of machine learning shows promise.

Artificial intelligence and in particular machine learning is achieving increasing utility in ophthalmology. Recent advances were made possible by incorporation of graphical processing units (GPU) into machine learning tasks [32, 33]. With large amounts of data available, some algorithms are now reporting advantages in diagnostics, and even in outcomes prediction for many prevalent conditions in ophthalmology. Notable among these are retina and glaucoma [34–37]. Despite potential benefits, for a number of applications of high interest, sufficiently detailed and categorized data is frequently not available. When data is insufficient, the developer may apply data analysis techniques such as cross-validation, ensemble and regularization in order to reduce the “overfitting”—the conditions from which the algorithm does not learn although it has “memorized” the data [38]. Despite these and other analytical tools, there are times when insufficient quality data is simply insufficient, and at other times it is simply not feasible to produce data on the requisite scale.

In addressing challenges with data availability, there are evolving developments in data acquisition, recording and display. Examples might include improved image quality as demonstrated by the difference between time domain Optical Coherence Tomography (OCT) images from 1991 [39] and the present swept source OCT quality of 2019. Novel image sources as provided by for example, increased adoption of “heads-up surgery” [40]—a 3D viewing system with an embedded 3D camera that records and is reproduced in a 4 K television. Other areas of data improvement include but are not limited to collection of data of higher quality, consistency, and availability. Further improvements in image quality, increasing data volumes, strategic categorization of data, are providing the foundations for next generation tools in ophthalmology and for the emergence of artificial intelligence/machine learning.

As a future perspective for robot-assisted eye surgery, camera image information combined with other sources of data such as intraoperative OCT images, robot end-effector position and force sensing measurements could improve the ability of a robot to assist or primarily perform selected tasks during surgery. Evolution of neural networks [41, 42], progress in image acquisition [43, 44] and a significant increase in data usage [45] may enhance the safety and effectiveness of robotic procedures, especially in eye surgery [46]. In addition to providing a new source of data, surgical viewing systems such as the “heads up surgery” systems, might also enable augmented reality during retinal procedures. Virtual reality systems such as Eyesi Surgical (VRmagic GmbH, Mannheim, Germany) and deep learning tracking algorithms could be used with robotic control and may help training, testing, and improving systems to increasingly avoid potential iatrogenic injuries. As improved data is the foundation of advancing artificial intelligence, enhanced safety, efficacy and increased reliability, are potential outcomes of incorporation of robotics into ophthalmology.

In conclusion, neither artificial intelligence nor robotics is a novel concept, until artificial intelligence is strategically incorporated into robotic systems. Many obstacles exist to human end user adoption of robotics including but not limited to cost, size, functional limits, accuracy, human acceptance and importantly, clearly superior outcomes and safety. Early historical concerns related to the role of the human in the decision-making process in robotic surgery has been largely put to rest, however recent developments in artificial intelligence applied to robotics may force the “overfitted” issue to be revisited. In retinal procedures, robotic platforms show a promising role and first human studies are encouraging. That artificial intelligence might enhance these systems is logical, the form that such augmentation takes is only now emerging. The road to feasibility of robotics augmented by artificial intelligence will meet a number of challenges, and there will continue to be a large and essential human roll most especially in the early stages of technology development. What the ultimate form will be is anyone’s guess, as is the eventual role of humans in microsurgery. For now however it is sufficient for engineers and surgeons to work cooperatively to develop cost-effective tools that improve patient safety, enhance procedure efficacy and extend surgical capability all for the betterment of patient care.

## Data Availability

Not applicable.

## Abbreviations

FDA: United States Food and Drug Administration
GPU: graphics processing unit
OCT: optical coherence tomography
